# Assessing catastrophic and impoverishing effects of health care payments in Uganda

**DOI:** 10.1186/s12913-015-0682-x

**Published:** 2015-01-22

**Authors:** Brendan Kwesiga, Charlotte M Zikusooka, John E Ataguba

**Affiliations:** HealthNet Consult, P.O. Box 35928, Kampala, Uganda; Health Economics Unit, School of Public Health and Family Medicine, University of Cape Town, Observatory, Cape Town, 7925 South Africa

**Keywords:** Financial health protection, Out-of-pocket payments, Catastrophic payments, Impoverishment, Uganda

## Abstract

**Background:**

Direct out-of-pocket payments for health care are recognised as limiting access to health care services and also endangering the welfare of households. In Uganda, such payments comprise a large portion of total health financing. This study assesses the catastrophic and impoverishing impact of paying for health care out-of-pocket in Uganda.

**Methods:**

Using data from the Uganda National Household Surveys 2009/10, the catastrophic impact of out-of-pocket health care payments is defined using thresholds that vary with household income. The impoverishing effect of out-of-pocket health care payments is assessed using the Ugandan national poverty line and the World Bank poverty line ($1.25/day).

**Results:**

A high level and intensity of both financial catastrophe and impoverishment due to out-of-pocket payments are recorded. Using an initial threshold of 10% of household income, about 23% of Ugandan households face financial ruin. Based on both the $1.25/day and the Ugandan poverty lines, about 4% of the population are further impoverished by such payments. This represents a relative increase in poverty head count of 17.1% and 18.1% respectively.

**Conclusion:**

The absence of financial protection in Uganda’s health system calls for concerted action. Currently, out-of-pocket payments account for a large share of total health financing and there is no pooled prepayment system available. There is therefore a need to move towards mandatory prepayment. In this way, people could access the needed health services without any associated financial consequence.

## Background

Globally, health systems are called upon to ensure universal access to health care for their populations [1]. This requires that they ensure the availability of adequate and quality health services for everyone in need of them while consequently protecting them from the accompanying financial burden [2]. The reliance on out-of-pocket payments for health care is a major cause of financial hardship in families. The World Health Report 2010 notes that over 100 million people are pushed into poverty while over 150 million people incur excessive out-of-pocket health payments that place a heavy drain on their living standards [1]. Despite the call for health systems to adopt sustainable health financing mechanisms as a path to universal coverage, most health systems in low income countries, particularly in sub-Saharan Africa, are still heavily dependent on out-of-pocket payments [3,4].

In Uganda, user fees were introduced in 1993 as part of a package of economic reforms recommended by the World Bank to reduce the level of debt and macroeconomic stagnation [5]. The rationale for these restructurings was based on a theoretical argument related to the price inelastic demand for health care. It was hoped that the health sector would be able to raise its own revenue so as to improve the quality of its services [5,6]. However, the implementation of the user fee policy not only failed to achieve these objectives, it also resulted in decreased health care utilisation consequently increasing morbidity and mortality [7,8]. In response, user fees were abolished in 2001. The abolition was expected not only to increase access to health care but to reduce the financial burden on households.

Indeed, public health care utilisation increased particularly among the poor after the removal of user fees [9-12]. However, among the rich, utilisation decreased to a rate even lower than before the elimination of the fees [12]. This is attributed to perceived differences in quality between the public and private sectors. Studies have revealed that the private health sector is the preferred provider for both the rich and the poor in Uganda [13-16]. The preference for private facilities which mainly rely on out-of-pocket payments has kept those payments high [17]. A further factor attributed to the high out-of-pocket payments is the presence of informal payments in the public health facilities [12]. It has been demonstrated that out-of-pocket payments as a percentage of private health expenditure in Uganda increased from 56.7% in 2000 to 64.8% in 2011 [18]. The most recent national health accounts exercise indicates that household contribution through out-of-pocket payments is the dominant source of health expenditure contributing about 50% of total health expenditure in the fiscal year 2009/10 [19].

Since illness is unpredictable, the consequent out-of-pocket payments incurred are likely to impose a financial burden on families. This is due to the impact of these payments on the allocation of the household’s disposable income. For instance, the outlay compromises the consumption of other household necessities such as food, clothing education and housing [20]. These payments may also lead to a decrease in welfare leading to an increase in the level of poverty [21,22]. It has been argued, therefore, that in a fair and equitable health system, households should not pay more than a certain proportion of their total income for health out-of-pocket [23,24]. Exceeding such a threshold would make such payments catastrophic. These payments should also not push families into poverty or exacerbate their existing state of poverty [23]. These arguments form the basis for the methodologies used in measuring financial protection in a health system.

Various earlier studies have investigated aspects of the financial burden of illness in Uganda [12,17,19,25-27]. A review of the literature indicates that these published studies did not quantify the overall impoverishing impact of out-of-pocket health care payments. Furthermore, in this paper, financial catastrophe is assessed using a recent and generalised methodology developed by Ataguba [24] that is able to demonstrate more concern for poorer socio-economic groups in society.

## Methods

### Data

The 2009/10 Uganda National Household Survey (UNHS) data is used for this study. This is a nationally representative survey conducted by the Uganda Bureau of Statistic (UBOS). The UNHS 2009/10 used the 2002 Uganda population and housing census sampling frame with a two-stage stratified sampling design to sample 6800 households. The first stage selects 712 enumeration areas (EAs). The EAs were allocated to ten sub-regions representing both urban and rural areas. In the second stage, ten households were systematically drawn from each of the selected EAs. UNHS data is available on the UBOS website (http://www.ubos.org/unda/index.php/catalog/51).

Data analysis utilises both ADePT version 5.2 software [28] and Stata version 11 [29]. All the estimates and the standard errors are adjusted for the sampling design using the appropriate sampling weights.

### Living standards and out-of-pocket measurements

Total household consumption expenditure is used as a proxy for income. Household consumption is a preferred measure because it is less prone to fluctuation and is easier to collect in household surveys with less likelihood of being underreported when compared to income [30]. The components of household consumption expenditure include food, beverages and tobacco, durable, semi durable and non-durable household goods, and other frequently purchased goods and services. Total income is converted into an adult equivalent household income so as to account for household composition. The adult equivalent scale used in this study has been used elsewhere see [31,32]. It is estimated as:

1 $$ AE=\left(A+\beta C\right) $$

where *A* represents the number of household members aged 18 years and above while *C* represents those below 18, *β* varies from 0.273 for the members below 1 year to 0.95 for members between 16 and 18 years.

Out-of-pocket payments used in this paper include consultation fees, medicines, facility charges and all the other health and medical costs not classified in the components above. These include expenditures on alternative/traditional medicines and fees.

### Assessing financial protection in a health system

#### Catastrophic out-of-pocket health care payments

Out-of-pocket health care payments are defined as catastrophic “if they exceed z% of household income (or resources) but with *z* increasing with income, that is, it is a rank-dependent threshold so that catastrophe is a function of where the individual, household or group sits in the income distribution range. Those at the upper end of the distribution face a greater proportion (threshold)” [24] p.314. The basic idea behind this reasoning is that such a threshold need not be the same for households of different socio-economic status as small out-of-pocket payments could be detrimental to those who are already poor. This method is a generalisation of that shown in [23]. In general, the methods developed in [24] may be used to replicate the results that use a constant threshold [23]. It also ensures that catastrophic impact is not only adjusted for vertical equity but is also able to adjust for the diminishing marginal utility of income.

If *y*_*i*_ is total household income, *T*_*i*_ is household total out-of-pocket payments and *Z*_*cat*_ is an initial threshold, then a rank dependent threshold ${Z}_{cat}^{\mathit{\hbox{'}}}$ can be defined as:

2 $$ {Z}_{cat}^{\mathit{\hbox{'}}}=w\left(p:\gamma \right)*{Z}_{cat} $$

where *γ* is a parameter of aversion to inequality, *p* is the household’s percentile and *w*(*p* : *γ*) = *γ*(1 − *p*)^(*γ* − 1)^ for *γ* ∈ (0, 1].

The restriction above implies that when *γ* =1, ${Z}_{cat}^{\hbox{'}}={Z}_{cat}$ as in [23] while when *γ* =0, ${Z}_{cat}^{\hbox{'}}$ is undefined. In this paper, following Ataguba [24], *γ* = 0.8 while the initial thresholds are (*Z*_*cat*_) = 5%, 10%, 15% and 25% of total household income.

Let ${O}_i^{\mathit{\hbox{'}}}$ represent the rank-dependent catastrophic overshoot (excess payment above a threshold) such that ${O}_i^{\mathit{\hbox{'}}}= \max \left(0,\left({T}_i/{y}_i\right)-{Z}_{cat}^{\mathit{\hbox{'}}}\right)$. If ${E}_i^{\mathit{\hbox{'}}}$ is a measure indicating whether a household exceeds the rank dependent threshold, then ${E}_i^{\mathit{\hbox{'}}}=1$ when ${O}_i^{\mathit{\hbox{'}}}>0$ and 0 otherwise. The rank-dependent headcount ratio (${H}_{cat}^{\mathit{\hbox{'}}}$) is defined as:

3 $$ {H}_{cat}^{\mathit{\hbox{'}}}=\frac{1}{N}\left({\displaystyle {\sum}_{i=1}^N{E}_i^{\hbox{'}}}\right)={\mu}_{E^{\mathit{\hbox{'}}}}^{\mathit{\hbox{'}}} $$

where ${\mu}_{E\mathit{\hbox{'}}}^{\mathit{\hbox{'}}}$ is the mean of *E* ' and *N* is the sample size. The headcount ratio measures the proportion of households that incur catastrophic payments.

The rank-dependent catastrophic gap (${G}_{cat}^{\mathit{\hbox{'}}}$) which captures the deviations from the catastrophic threshold ${Z}_{cat}^{\mathit{\hbox{'}}}$, is computed as:

4 $$ {G}_{cat}^{\mathit{\hbox{'}}}=\frac{1}{N}\left({\displaystyle {\sum}_{i=1}^N{O}_i^{\mathit{\hbox{'}}}}\right)={\mu}_{O\mathit{\hbox{'}}}^{\mathit{\hbox{'}}} $$

where ${\mu}_{O\mathit{\hbox{'}}}^{\mathit{\hbox{'}}}$ is the mean of the overshoots (${O}_i^{\mathit{\hbox{'}}}$).

#### Impoverishment impact of out-of-pocket payments

This is the increase in poverty that results from household’s incurring out-of-pocket costs for health care [23]. If *PL*_0_ is the poverty line and *Y*_*i*_ is individual *i*’s prepayment adult equivalent household income, an individual is poor (i.e., prepayment poverty (${P}_i^{pre}$)) if *Y*_*i*_ < *PL*_0_. Therefore, prepayment poverty headcount ratio is computed as:

5 $$ {H}_{pov}^{pre}=\frac{1}{N}{\displaystyle {\sum}_{i=1}^N{P}_i^{pre}}={\mu}_{p^{pre}} $$

The short-fall from the poverty line (${g}_i^{pre}$) is defined as (*Y*_*i*_ − *PL*_0_) if *Y*_*i*_ < *PL*_0_, and zero otherwise. The associated average prepayment poverty gap is defined as:

6 $$ {G}_{pov}^{pre}=\frac{1}{N}{\displaystyle {\sum}_{i=1}^N{g}_i^{pre}}={\mu}_{g^{pre}}. $$

The normalised poverty gap is computed as:

7 $$ N{G}_{pov}^{pre}={G}_{pov}^{pre}/P{L}_0. $$

When the superscripts “pre” are replaced with “post” the analogous post-payment poverty measures are obtained. The poverty impact of out-of-pocket payment is then defined as the difference between the relevant pre-payment and post-payment measures. In order to compute the percentage change in poverty as a result of out-of-pocket payments, relative ratios are obtained by dividing the difference between the prepayment and post payment poverty measures by the prepayment poverty measures.

Uganda’s poverty line (PL1) which is region and location specific and the World Bank $1.25 per day international poverty line (PL2) are used in this study. The maximum value of PL1 is in central urban (Shs. 32106.24 per month) while the least is for western rural (Shs. 28165.4 per month). The average value is Shs. 29,306.32 per month. Based on the Purchasing Power Parity (PPP), the $1.25/day poverty line is equal to Shs. 27,923.18 per person per month.

Pen’s parade of ‘dwarfs and a few giants’ that plots two income parades (i.e., gross income and income net of out-of-pocket payments) using a cumulative proportion of individuals ranked according to their gross household income [33], is also used in this paper. It illustrates the welfare decreasing effect of the out-of-pocket payment by showing the increase in the extent and depth of poverty as a result of such expenses.

Uganda’s poverty line (PL1) which is region and location specific and the World Ethical clearance for the study was obtained from the Uganda National Council of Science and Technology (REF: SS 2463) and the Health Research Ethical Committee (HREC) of the University of Cape Town (REF: 248/2012).

## Results

### Household catastrophic out-of-pocket health payments

As indicated in Table 1, a large number of households spend a substantial share of their total income on out-of-pocket health care. The proportion of households that spend above the thresholds varies depending on the initial threshold. With a 5% initial threshold for example, the catastrophic headcount ratio is estimated at 38%. This represents over two million households based on the population estimate of about six million households in the UNHS 2009/10. The catastrophic headcount ratio decreases to 22.8% at the 10% initial threshold.

**Table 1:** **Household catastrophic out-of-pocket health payments results for variable thresholds**

	**Initial thresholds**			
	**5%**	**10%**	**15%**	**25%**
**Headcount ratio**	38.0%	22.8%	15.3%	6.7%
**Gap**	3.8%	2.5%	1.7%	0.8%

*Source*: Authors’ computations based on UNHS 2009/2010.

*Note*: The parameter of aversion to inequality, *γ* = 0.8.

As also demonstrated in Table 1, the catastrophic health payments gap varies from 3.8% (at the 5% initial threshold) to 0.8% at an initial threshold of 25%. This indicates that not only is the number of households that incur catastrophic out-of-pocket payments (defined by exceeding those thresholds) high, but households also substantially exceed these thresholds.

### Impoverishment effect of out-of-pocket payments

As demonstrated in Table 2, using the Ugandan poverty line, out-of-pocket payments led to a 4.2% rise in poverty head count ratio. This represents over one million more Ugandans being pushed below the poverty line. The normalised poverty gap increased by about 1.4% representing about 21% relative increment. A similar pattern is observed using the World Bank $1.25 a day poverty line (see Table 2).

**Table 2:** **Impoverishment analysis results UNHS 2009/2010**

	**PL 1 (Uganda’s poverty line)** ^**a**^			**PL 2 ($1.25)**		
	**Gross (A)**	**Net (B)**	**Relative (B-A)/(A)**	**Gross (C)**	**Net (D)**	**Relative (D-C)/(C)**
Headcount ratio	24.5%	28.7%	17.1%	22.7%	26.8%	18.1%
Normalised gap	6.7%	8.1%	20.9%	6.3%	7.6%	20.6%

*Source*: Authors’ computations based on UNHS 2009/2010.

*Note*: ^a^Average poverty line is Shs. 29,306.32 ($1.31) per month.

**Figure 1:**
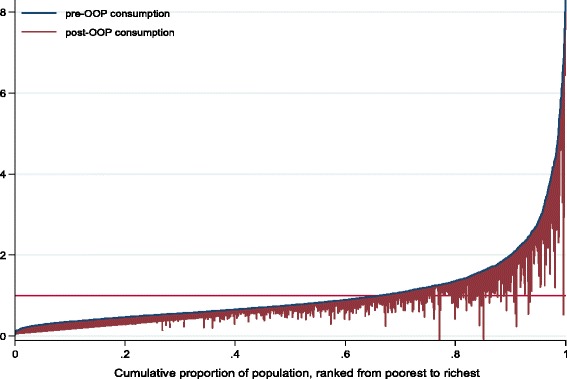
**Pen’s parade of household consumption gross and net of out-of-pocket payments.** Source: Authors computations based on UNHS 2009/10.

The Pen’s chart in Figure 1 indicates that out-of-pocket payments lead to a significant decrease in household welfare even increasing the extent of poverty among the currently non-poor. This is indicated by the decrease in household consumption expenditure shown by the “paint drips”.

## Discussion

The results indicate a lack of financial protection in Uganda’s health system. A very high percentage of Ugandan households incur financial catastrophe irrespective of the threshold considered and these out-of-pocket payments impoverish about 4% of Ugandans representing over 17% relative raise in poverty in the country. Since out-of-pocket health care payments account for a substantial share of household budget, they are likely to compromise the consumption of other basic necessities [20,34].

The results relating to financial catastrophe in this paper follow similar patterns to those of previous studies around the financial burden of out-of-pocket payments in Uganda and elsewhere [12,24,27,35,36]. Although, to the knowledge of the authors, no previous studies in Uganda have analysed the impoverishment effect, the findings in this paper are similar to those reported in multi-country and other related studies [25,37-40]. Higher levels of impoverishment have been reported in other African countries especially those which, like Uganda, depend heavily on private health financing through out-of-pocket payments [41-44].

It is possible that the impact of such payments in terms of both financial catastrophe and impoverishment reported in this paper is understated. This is because the poor may be too poor to even afford falling ill and therefore modify their perception of illness so as to avoid incurring payments [22]. It may also be argued that since the poor utilise more free public facilities than the rich [12], their exposure to out-of-pocket payments is decreased. In that case, the impact is not understated. However, since the demand for public sector facilities is lower than that for private sector facilities as indicated by the preference for the latter [13,15,16], it is only those who can afford to pay for the private sector services that utilise them.

The lack of financial protection as revealed in this paper has important implications for the population. There is, therefore, a need for the country to limit direct payments that impose burdens on households. Based on the international literature [1], this may take the form of moving towards prepayment (particularly mandatory) for health care as a means of attaining universal coverage. In low and middle income countries, mandatory prepayment has been shown to increase the level of financial protection [45-49]. For the private facilities where fee for service is the dominant provider payment mechanism, high out-of-pocket payments can be reduced by adopting a provider payment mechanism that does not increase household out-of-pocket expenditures.

This study has some limitations. The adjustment for vertical equity and diminishing marginal utility in the measurement of catastrophic payments as presented in this paper uses the inequality aversion parameter. The choice of the value of this parameter is still subjective [24]. Ideally, this parameter should be guided by the community’s weighted preferences representing how compassionate a society is toward the poor [24]. It is still the case that the use of variable thresholds provides an indication of the manner in which societies view consideration of equity and fairness, as a small out-of-pocket payment by the very poor could be far more financially catastrophic. Furthermore, the study does not identify household characteristics that increase the likelihood of incurring both catastrophic payments and impoverishment. These are some of the areas on which, particularly in Uganda, further research on financial risk protection in a health system should focus.

## Conclusion

The absence of financial protection in Uganda’s health system calls for concerted action aimed at reducing the large proportion of out-of-pocket payments currently present in total health financing. The results indicate that Uganda, like many other African countries, is far from attaining the kind of universal health coverage which would emphasise protection of the poor especially. There is a need to put in place pooled prepayment systems so as to place the country on the road to achieving universal health coverage where people do not face financial difficulties in accessing the necessary health services.
